# Facts and Fancies in Orthopaedics

**Published:** 1968-10

**Authors:** Norman Capener


### FACTS AND FANCIES IN ORTHOPÆDICS

Kenneth Pridie Memorial Lecture

TRURO, 13th May, 1967

BY

NORMAN CAPENER

Many men and women in the South West of England will remember Kenneth Pridie with affection and admiration, in particular those surgeons who will think of his courage when he died while addressing them at an orthopædic meeting at Exeter on the 4th May, 1963. He was a humane and friendly man who worked and spoke with determination and a high sense of duty which permitted no compromise. With all his skills Pridie was a pioneer in the field of bio-mechanics in the modern sense, for he stressed the importance of rigorous tests of the performance of metallic and other implants under the conditions of engineering workshop practice.

[In reviewing Pridie's work, the lecturer dealt with the history of the use of mechanical devices in the treatment of locomotor disease and injury from the time of Hippocrates and the ancient armourers to modern times, and in particular with the work of Hey Groves whom Pridie held in such high regard. In this matter it seemed appropriate to outline some of the recent work carried out at the British Standards Institution in the matter of materials and design for metal surgical implants.]

### STANDARD METALS AND DESIGN

In the British Standard, materials for implants have been specified as the most suitable, in the light of present experience, for providing the best mechanical properties, resistance to corrosion, and physiological compatibility with living tissues. In the case of implants made of metal, it is important to ensure that the components are made of material of the same composition. The ideal material has yet to be found. It should be electrolytically inert in the body, free from corrosion in body fluids, readily available, easy to fashion by usual engineering methods, of adequate strength for the forces encountered, and economically acceptable.

The search for the ideal metal goes on. Each one of those in the British Standard has both advantages and disadvantages. We need strength, hardness and lightness with complete freedom from corrosion and, if possible, a metal which is freely available in this country for fabrication by British craftsmen. It is possible, according to Mears, that titanium alloyed with molybdenum may provide this but as yet this is not proved by biological tests.

Although corrosion has been almost completely eliminated in the surgical use of British Standard steel of chrome-nickel-molybdenum, it may occur in a relatively minor form as fretting or contact corrosion for the following reasons. It is necessary to maintain on the surface of this metal a high polish which is associated with a passivation of the metal and the production of a surface film of chromium oxide. This oxide must be maintained by free access to oxygen such as is available in the tissues of the body. When two metals are in contact the crevice between them does not allow this freedom of oxygenation. A further aggravation may be produced by friction or fretting if there is movement between the sections. This causes a breakdown of the chrome oxide metallic film.

One of the important achievements of the British Standard for Surgical Implants was the design of the screw, the most frequently used single implant. We owe a particular debt to Dr. J. T. Scales for all the work he did on this, the details of which still await publication though completed at least 6 years ago. In the Standard a move has been made towards the metric system in devising the 4 millimetre screw (the equivalent of 5/32 of an inch). The core diameter of this screw, upon which so much of its strength depends, being 13% greater than that of the 9/64 inch screw, has almost 50% greater increase in the mean torsional yield stress. There is obviously a limit to the size of screws. One has felt that larger diameters might lead to breakage of the bones through which they pass. Allgower and Müller have shown, however, that a larger screw can be used. Nevertheless, the British screw properly inserted does have fully adequate stress resistance. There are, however, as Scales has shown, matters of surgical technique in the insertion of screws that require much more serious consideration than is usually given. Briefly they relate to the type of screw, whether self-tapping or otherwise; the type of screw driver and the character of the bone being treated. The main thing is that if a force is applied to the screw greater than its torsional yield strength it will tend to break. This may be due to the hardness of the bone. Tests of the character of bone in different individuals show that this is variable within wide limits. For example, Scales found that a self-tapping 4mm screw inserted in one cortex of a human femur required a force of $\frac{3}{4}$ of a ton to pull it out, while in another bone the same type of screw could be pulled out with only just over 1/10 of a ton. A self-tapping screw is easier to insert but, because of the ingrowth of bone into the flutes, is more difficult to remove than a non-self-tapping screw. In its removal a torque stress may be applied by a surgeon's hand exceeding its yield point and so it may break. The other type of screw, without flutes, for the same reason may break, or suffer fatigue during insertion which may lead to later breakage. It is clear that the surgeon should use normal engineering practice by tapping drill holes to receive a non-self-tapping screw before its introduction. Furthermore there is adequate strength if the screw, passing through a hollow diaphysis, has to enter a thread only in the distal cortex, the proximal cortex having been reamed to a larger diameter. Thus will be avoided the distraction force which increases torque stress when both holes are tapped to take the screw. Scales has shown that screw drivers should be of a type that provide a limitation of torque strength to say 45 lb. per square inch; which is less than is possible with the usual types of screw driver and is adequate if drill holes are tapped before insertion.

One other point arises from the studies of screw insertion. If force is applied intermittently (as with a hand screw driver) then excess torque tends to be exerted with the influence of the inertia arising before each movement. There is strong argument for inserting screws with continuous rotation by the use of a brace which will hold the screw driver bit.

In brief, then, non-self-tapping screws should be used through a pre-tapped drill hole of the right dimension, the screw holding only in the tapped distal cortex and inserted by a torque-limiting screw driver driven continuously at slow speed.

What must be remembered is that bone is not a static material. Not only does it vary in quality from one person to another; it also changes its character from week to week according to the circumstances of life of the individual and probably more rapidly after injury. As is apparent from recent metabolic studies, after any injury of magnitude, there is rapid general loss of protein from the body. This is not simply due to immobilisation. The protein content of bone is $\frac{1}{3}$ of its weight and $\frac{2}{3}$ of its volume, it is therefore understandable that osteoporosis quite apart from the influence of a foreign body will seriously affect the strength of hold of any material used for osteosynthesis.

### VESTED INTERESTS AND ORIGINALITY

In medicine the days are almost past when financial considerations lead these interests to be personal and surrounded with secrecy. In the pharmaceutical world it is true that, following American practice, advertising and patenting have become powerful. In surgery, those who practise it tend to look askance at those practitioners whose therapeutics are aligned too closely to their financial gain. A less pernicious aspect, however, is even more prevalent and few surgeons at some time or another are not guilty of it; the application of surgical techniques with diminishing self-criticism—a technique learnt from a senior or developed personally. A Cartesian philosophy of perpetual doubt is difficult to maintain in a world of increasing pressure and in an individual who is steadily ageing—as every consultant is.

All practitioners have a vested interest against time and boredom. It is easier, for instance, with a patient suffering from spinal strain, to prescribe a surgical corset and so get rid of him—but is he cured? Why not a manipulation on the examining couch, a few strong words about exercise and getting on with the job, and perhaps a temporary workman's belt or binder?

[The lecturer then dealt with his own vested interests and prejudices discussing his interests in Potts paraplegia, lively splintage, the use of plastics in joint reconstruction and fixation devices for pertrochanteric fractures.]

The amassing of large series of operations has elements of vested interest if it is not controlled by critical judgment; and there are two sides to this. When in 1954 I brought scorn upon myself by issuing a caution upon the failure of the Judet arthroplasty, I happened to be in correspondence with Geoffrey Jefferson. I asked him what he thought of large series of operations for judgment of success. He said that 50 examples of any experiment was sufficient. " After that " he said " experience may be merely the repetition of the same error." Or as Hippocrates said " Art is long; life is short; experience is fallacious."

For a surgeon studying clinical results the second element of fallacy is the failure of critical judgment in the assessment of a procedure for which he has been responsible, in the presence of the patient who does not wish to offend him.

We all tend to pour scorn on osteopaths, bone-setters and other empirics. It is right that we should maintain our scepticism, but we might look well to ourselves. We should probably find that quite often we prescribe treatment or give advice for which there is no strictly scientific support. Do we always know precise causes? Why are we so ready to accept the latest propaganda or the newest-fangled therapeutics?

Harry Platt in one of his writings has said something about fundamental principles or ideas and has remarked how few they are, and how ancient. Advances tend so often to be in the fields of technology. Mouth to mouth respiratory resuscitation is said to have been used by Elijah. It was certainly advocated by a Naval surgeon in the beginning of the 19th century long before the recent rediscovery of the technique by Lindahl of Norway.

Denis Browne's ideas upon club foot and its attribution to intra-amniotic pressure is at least as old as Hippocrates. It was illustrated by Parker and Shattock in 1877. The principle of his hobble splint was illustrated by Sayer in the mid-19th century and the principle was also used by the early Italian ballet masters with the mistaken idea of bandaging their pupil's legs to boards at night to encourage eversion of the hips. The Americans think that Buck invented the use of continuous skin traction in the treatment of femoral shaft fractures but this was first published by J. H. James in 1832. The Smith-Petersen nail for femoral neck fractures was certainly a technical advance, but the idea was an old one. In Amsterdam 41 years ago, one saw Noordenbos the Senior pinning femoral neck fractures with lengths of fibula—the perfect tri-fin autograft. The method apparently was devised by another Dutchman, Metz, that inventive genius in fracture treatment, who (amongst other methods) devised the Balkan beam.

[The lecturer completed his address with a brief historical note in honour of a group of visiting surgeons from the Netherlands. This dealt with the influence of Dutch workers in medicine during the late 17th century and particularly the artists and printers who did so much to raise the standard of medical literature.]

